# Perinatal dengue in a neonate with multiple comorbidities: case report and literature review

**DOI:** 10.3389/fped.2025.1685280

**Published:** 2025-10-27

**Authors:** Guanghong Li, Xiaoqun Du, Weibin Luo, Junhua Wei, Huiyi Huang

**Affiliations:** Department of Pediatrics, Huadu District People’s Hospital of Guangzhou, Guangzhou, China

**Keywords:** neonatal dengue, maternal vertical transmission, meconium aspiration syndrome, neonatal pneumonia, neonatal anemia

## Abstract

**Background:**

Dengue is an increasingly common arboviral infection in endemic regions and can affect pregnant women. Neonatal presentations are uncommon and often overlap with other perinatal conditions such as meconium-related lung disease and early-onset bacterial infection, complicating diagnosis and management.

**Case presentation:**

We report a term male neonate born through meconium-stained amniotic fluid to a mother with late-pregnancy dengue. Within hours, the infant developed tachypnea and coarse rales. Initial studies showed metabolic acidosis and coagulopathy; chest radiography demonstrated bilateral patchy opacities. A working diagnosis of meconium aspiration syndrome was made, and empiric penicillin, oxygen, and supportive care were initiated. On day 2, dengue NS1 antigen tested positive; Respiratory PCR detected low-level *Ureaplasma urealyticum*, though its clinical significance was uncertain. With maternal dengue and compatible clinical features, neonatal dengue was clinically diagnosed, while perinatal transmission was considered likely but not definitively proven in the absence of neonatal RT-PCR or paired serology. From days 5–10, the infant developed fever and marked thrombocytopenia (nadir 29 × 10⁹/L) without hemodynamic instability. Intravenous immunoglobulin (1 g/kg) was given for immune modulation, and a short azithromycin course targeted atypical bacteria. By day 14, respiratory findings, inflammatory markers, and platelet count improved, and the infant was discharged in stable condition with arranged follow-up.

**Conclusions:**

Neonatal dengue should be considered in infants born to mothers with recent dengue in endemic settings. A structured approach—early supportive care, targeted testing (NS1/RT-PCR/serology), careful interpretation of neonatal coagulation and platelet trends, and judicious antimicrobial use—can aid differentiation from overlapping perinatal lung disease and guide timely management.

## Introduction

Dengue fever, a mosquito-borne viral disease (1), has emerged as the fastest-growing global arboviral infection, with its annual incidence escalating more than eightfold over the past two decades. According to the World Health Organization (WHO), surveillance systems have recorded over 7.6 million suspected dengue cases worldwide as of April 30, 2024, including 3.4 million laboratory-confirmed infections, 16,000 severe cases, and more than 3,000 dengue-related deaths (2). Dengue is caused by four antigenically distinct serotypes (DENV-1 to DENV-4) and is transmitted primarily by Aedes aegypti and Aedes albopictus mosquitoes; humans are the main amplifying hosts (3). Diagnostic modalities include NS1 antigen detection and RT-PCR during the early viremic phase, followed by serology (IgM/IgG) in later stages, each with time-dependent performance characteristics (4). Neonatal dengue infections are uncommon in surveillance and literature reports, and their true frequency is likely underestimated due to diagnostic challenges and nonspecific presentations. In neonates, acquisition is most often linked to maternal infection near delivery, via *in utero* (transplacental) or intrapartum exposure; transfusion-related transmission is rare but possible (5). Reported transmission likelihood varies by study and is influenced by the timing/intensity of maternal viremia and placental factors; thus, point estimates should be interpreted cautiously.

In China, particularly in southern regions such as Guangzhou and the Pearl River Delta, dengue resurgence has been epidemiologically documented. Surveillance reports from Guangzhou revealed 437 confirmed cases in 2024, reflecting an escalating annual incidence trend (6). These localized epidemic patterns suggest heightened neonatal infection risks during peak transmission seasons, particularly among infants born to mothers with gestational dengue exposure. Neonatal aspiration syndrome (NAS), characterized by meconium or amniotic fluid inhalation, represents a prevalent respiratory disorder in newborns (7). Its clinical spectrum ranges from transient tachypnea to life-threatening respiratory failure, with aspiration pneumonia constituting the most frequent complication. Given overlapping early signs between neonatal respiratory disorders and systemic infections, a structured, age-appropriate diagnostic approach is essential. In endemic settings, early consideration of neonatal dengue is warranted when evaluating term infants with respiratory distress and evolving cytopenias/hemostatic abnormalities, particularly with maternal dengue near delivery. Although typically attributed to singular etiological factors, emerging clinical evidence highlights the coexistence of NAS with polymicrobial infections, including viral and bacterial pathogens. Such coinfections exacerbate systemic complications, including myocardial injury, thrombocytopenia, and coagulopathy, necessitating early diagnostic intervention and multimodal respiratory support.

The clinical management of neonatal dengue presents unique challenges. First, immunological immaturity in neonates manifests as nonspecific symptomatology—respiratory distress, feeding intolerance, and lethargy—that overlaps substantially with other neonatal pathologies, complicating timely diagnosis. Moreover, maternally derived dengue-specific antibodies can influence disease expression and complicate serologic interpretation, and may affect severity through immunologic mechanisms such as antibody-dependent enhancement (ADE). Typical laboratory features include thrombocytopenia and variable coagulopathy; clinicians should interpret these using neonatal reference ranges and serial trends. Prior reports have documented perinatal transmission leading to neonatal illness, reinforcing the need to consider dengue in symptomatic neonates born to mothers with recent dengue infection (8). Among published neonatal cases, diagnosis has relied on combinations of NS1 and/or RT-PCR positivity in the infant, maternal–infant serologic profiles, and compatible clinical courses.

Concomitant perinatal factors and potential copathogens (e.g., bacterial or mycoplasmal organisms) may modulate clinical severity, though definitive mechanistic synergy is difficult to establish in routine care. Accordingly, careful microbiologic evaluation, judicious antimicrobial use, and age-specific interpretation of hematology and coagulation parameters are recommended.

Here, we report a neonatal dengue case with multiple comorbidities managed at our center, occurring in the setting of meconium-stained amniotic fluid and maternal dengue during pregnancy. We detail the diagnostic reasoning, longitudinal clinical course, and management decisions, and discuss how perinatal factors and possible coinfections may intersect with neonatal dengue presentation. Our aim is to provide practical insights for clinicians in endemic regions in structuring evaluation and supportive management of similar cases, rather than to assert novelty based on patient age.

## Case presentation

### Patient information

This report describes a male neonate born via spontaneous vaginal delivery at Guangzhou Huadu District People's Hospital on November 11, 2024, at 05:22 AM. The infant was delivered at a gestational age of 40 weeks + 3 days, with a birth weight of 2.8 kg. Apgar scores at 1, 5, and 10 min were all 10. The neonate's length was 48 cm, head circumference 32 cm, and chest circumference 31 cm. The mother (Ms. Xie, 28 years old, an accountant) was diagnosed with dengue fever in late pregnancy, with a peak fever of 39°C during the course of illness, and was concurrently diagnosed with group B Streptococcus (GBS)-associated vaginitis. Her pregnancy was otherwise uncomplicated, with no history of antenatal anomalies detected on routine ultrasounds or laboratory tests; this was her first pregnancy and delivery (gravida 1, para 1), with no prior miscarriages, preterm births, or other obstetric complications. The maternal dengue and GBS vaginitis may have posed risks for perinatal infection and potential congenital abnormalities, such as low birth weight or respiratory compromise, though no structural anomalies beyond a patent foramen ovale were identified in the neonate. She did not receive antiviral or antibiotic treatment during pregnancy and was managed with supportive care. During delivery, the amniotic fluid was classified as Grade III meconium-stained, and the umbilical cord was wrapped around the neck once. The placenta appeared morphologically and functionally normal.

Shortly after birth, the neonate developed rapid breathing (respiratory rate: 56 breaths/min) with audible moist rales but showed no cyanosis, seizures, or apnea. The initial body temperature was 36.6°C. Physical examination revealed a head circumference of 32 cm, with a firm, 4 × 3 cm caput succedaneum that crossed suture lines but had no fluctuance. Auscultation of the lungs revealed coarse breath sounds with scattered coarse wet rales. Cardiac examination showed a heart rate of 140 beats/min, regular rhythm, and no pathological murmurs. Abdominal palpation detected a soft liver edge 1 cm below the right costal margin, with no palpable splenomegaly. Neurological assessment demonstrated normal muscle tone in all four limbs, with intact rooting, grasp, Moro, and sucking reflexes.

The infant's parents were both from Meizhou, Guangdong Province (Han ethnicity), and both had blood type B, Rh-positive. There was no family history of hereditary diseases or consanguinity. This case is notable for the coexistence of maternal dengue fever and bacterial vaginitis, potentially leading to fetal exposure to multiple pathogens via the placenta or birth canal, thereby contributing to a complex postnatal clinical course. The potential impact of maternal co-infections on neonatal presentation was considered during ongoing evaluation.

### Ethics statement

This case report was conducted in accordance with the ethical standards of the Declaration of Helsinki and its later amendments. The publication of this case report was reviewed and approved by the Ethics Committee of Huadu District People's Hospital (approval number: HDPH-2025-076). Written informed consent was obtained from the patient's parents (legal guardians) for the publication of this case report, including all clinical details and accompanying images. All patient information has been de-identified to protect privacy while maintaining scientific integrity. The parents were informed about the purpose of the publication and potential benefits to medical knowledge, and they were assured that their child's care would not be affected by their decision regarding participation in this report.

### Diagnostic assessment

Based on the clinical findings, laboratory results, and imaging studies, the patient's diagnoses were refined as:

1. Dengue fever (neonatal illness with positive NS1 antigen; perinatal acquisition suspected based on maternal infection history)
2. Meconium aspiration syndrome
3. Neonatal caput succedaneum
4. Neonatal bacterial pneumonia (possible; Ureaplasma spp. detected in upper airway—pathogenic role uncertain)
5. Neonatal ischemic-hypoxic myocardial injury
6. Patent foramen ovale

These diagnoses were supported by distinct diagnostic modalities: dengue fever was confirmed by a positive NS1 antigen test (maternal and neonatal serum) and clinical correlation; meconium aspiration syndrome was supported by characteristic findings on chest radiography; neonatal bacterial pneumonia was suspected based on respiratory pathogen PCR detecting *Ureaplasma urealyticum* from upper airway specimens; ischemic-hypoxic myocardial injury was inferred from transient elevation of myocardial enzymes and echocardiographic findings; and a patent foramen ovale was identified by echocardiography. These integrated findings guided the initial treatment, which included antibiotic therapy, respiratory support, and monitoring for potential complications.

### Clinical progression and treatment adjustment

#### November 11, 2024

**Figure 1:**
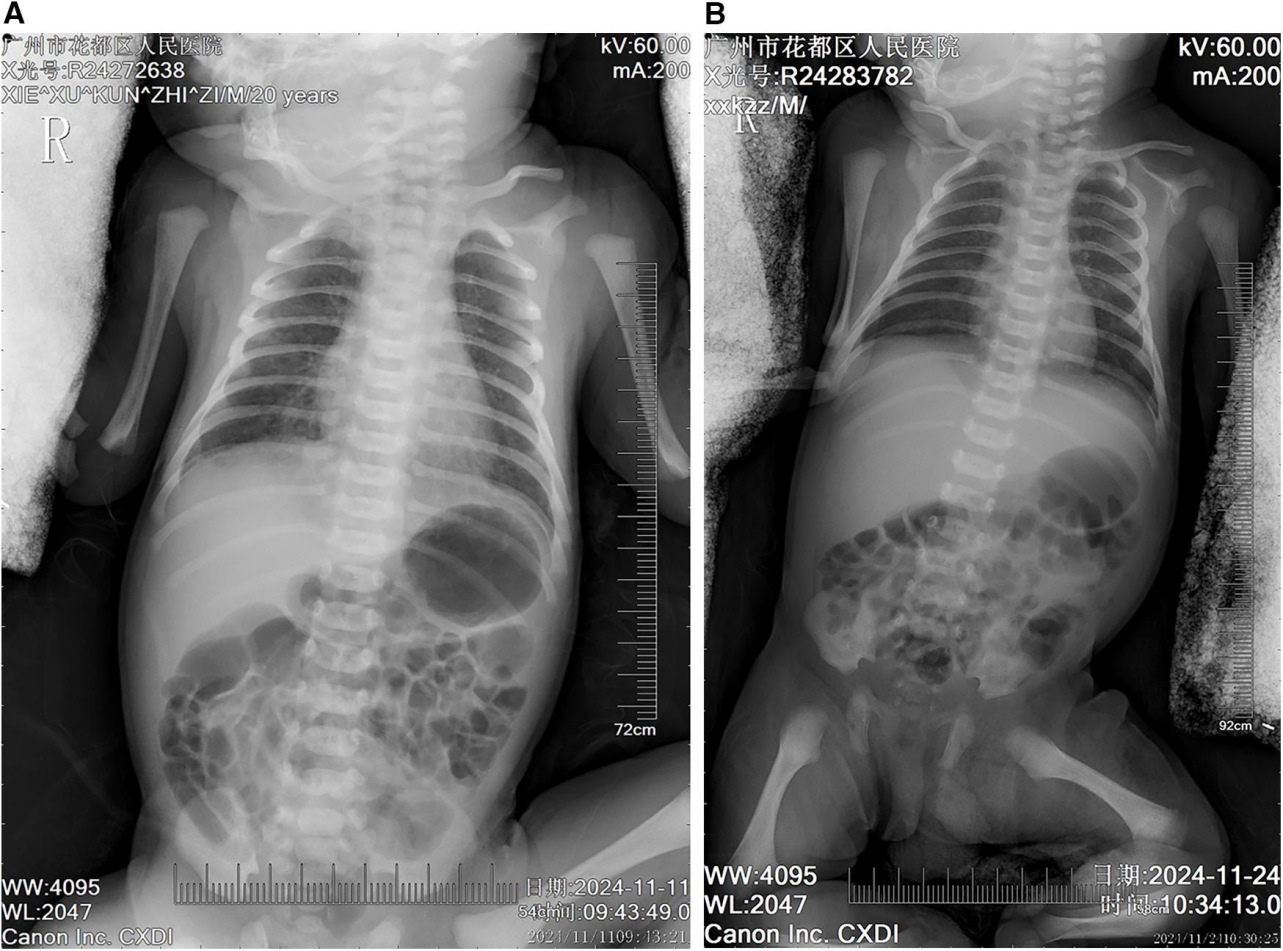
Chest radiographic changes in a neonate with meconium aspiration syndrome before and after treatment **(A)** initial chest radiograph (day 0 of life) showing diffuse bilateral pulmonary infiltrates with hyperinflation, patchy opacities, and flattened diaphragm consistent with meconium aspiration syndrome. Note the increased lung markings and coarse reticular pattern throughout both lung fields. **(B)** Follow-up chest radiograph (day 14 of treatment) demonstrating significant improvement in pulmonary aeration with resolution of the diffuse infiltrates. The lung fields appear clearer with normal lung volumes and diaphragmatic contour, indicating successful response to respiratory support and antimicrobial therapy.

The patient, a neonate, was admitted to the Department of Neonatology at 06:37 on November 11, 2024, due to “grade III meconium-stained amniotic fluid, and tachypnea and rales for over an hour after birth.” Upon admission, based on the grade III meconium-stained amniotic fluid and postnatal respiratory distress, a preliminary diagnosis of meconium aspiration syndrome (MAS) was made, and caput succedaneum was noted. Chest x-ray (Figure 1A) revealed increased and thickened bilateral lung markings, with patchy, ill-defined opacities in the lung fields, predominantly in the right lower lobe, suggestive of neonatal aspiration pneumonia. Cardiac ultrasound demonstrated a patent foramen ovale (PFO, 2.9 mm) and mild tricuspid regurgitation, with no other significant structural cardiac abnormalities detected. Initial laboratory investigations indicated coagulopathy, evidenced by elevated D-dimer (2,280.46 ng/mL), decreased fibrinogen (1.53 g/L), prolonged prothrombin time (PT 18.8 s), prolonged activated partial thromboplastin time (APTT 55.1 s), and metabolic acidosis (pH 7.314, lactate 4.80 mmol/L). Initial treatment included intravenous penicillin (50,000 units/kg every 12 h) for empirical coverage of common neonatal bacterial infections, oxygen supplementation to maintain SpO₂ > 90%, intravenous infusion of 10% glucose solution for blood glucose stabilization, and a single intramuscular injection of vitamin K1 1 mg for prophylaxis against hemorrhagic disease of the newborn. Supportive care and close monitoring were prioritized given diagnostic uncertainty on day 1.

#### November 12, 2024

On the second hospital day, new findings emerged. A multiplex respiratory PCR panel using a nasopharyngeal aspirate (NPA) specimen detected low-level *Ureaplasma urealyticum* DNA (9.78 × 10^2^ copies/mL), which was interpreted as possible colonization. In parallel, dengue NS1 antigen was positive in both maternal and neonatal serum by a rapid immunochromatographic assay; confirmatory neonatal RT-PCR was not performed due to low expected viremia and logistical constraints. Taken together with maternal dengue in late pregnancy and compatible neonatal features, these results supported a clinical diagnosis of neonatal dengue; perinatal transmission was considered likely but not definitively proven in the absence of neonatal RT-PCR or paired serology. Inflammatory markers increased (CRP 25.70 mg/L, WBC 22.31 × 10⁹/L, neutrophils 87.50%). A Th1/Th2 cytokine panel showed markedly elevated interleukin-6 (IL-6) 113.56 pg/mL, consistent with a systemic inflammatory response. Given Ureaplasma detection from the upper airway—where colonization is common—the pathogenic role was considered uncertain; azithromycin 10 mg/kg/day for 3 days (November 14–16) was added to cover atypical pathogens, with intensified daily monitoring of respiratory status and inflammatory markers.

#### November 15–20, 2024

During the 5th to 10th days of hospitalization, the neonate developed fever, with a maximum temperature of 38℃. Repeat complete blood count revealed a significant decrease in platelet count, reaching a nadir of 29 × 10⁹/L on November 20, 2024. Repeat coagulation tests showed persistently elevated D-dimer levels (1,324.22 ng/mL), fluctuating low fibrinogen levels (1.55 g/L), and a prolonged activated partial thromboplastin time again (82.2 s). Repeat cardiac ultrasound showed a slight reduction in the size of the patent foramen ovale (2.6 mm), with no significant change in the degree of tricuspid regurgitation, and no new structural cardiac abnormalities. Blood culture results were negative, initially ruling out bacterial sepsis. Given marked thrombocytopenia in the setting of suspected neonatal dengue, the treatment team decided to administer intravenous immunoglobulin (IVIG) 1 g/kg from November 20 to 21, 2024, to modulate immune function and support platelet recovery. Simultaneously, as the patient's SpO₂ remained stable above 94% at a lower oxygen flow rate, the oxygen flow was reduced to 1l/min.

#### November 24, 2024

On the 14th day of hospitalization, the patient's condition significantly improved. Repeat chest x-ray (Figure 1B) showed near resolution of bilateral pneumonia. Laboratory investigations revealed a marked decrease in inflammatory markers, with CRP decreasing to 0.25 mg/L, WBC to 13.19 × 10⁹/L, D-dimer to 973.79 ng/mL, and platelet count recovering to 140 × 10⁹/L. Repeat arterial blood gas analysis demonstrated normalization of acid-base balance (pH 7.35, partial pressure of carbon dioxide 34.70 mmHg, base excess −1.6 mmol/L), good oxygenation (partial pressure of oxygen 75.20 mmHg), and normalization of lactate levels (2.10 mmol/L). With comprehensive assessment indicating clinical improvement, penicillin and azithromycin were discontinued, and low-flow oxygen (1l/min) and nutritional support were continued, along with close monitoring of respiratory status, jaundice levels, and platelet count. No bleeding manifestations or hemodynamic instability were observed prior to discharge.

#### November 25, 2024

After 14 days of hospitalization, the patient's condition stabilized, meeting discharge criteria. At discharge, the clinical condition was good, afebrile, with stable respiration (spontaneous respiratory rate <60 breaths/min), resolved caput succedaneum, and only mild jaundice (transcutaneous bilirubin TCB 52 μmol/L). Laboratory tests showed total bilirubin at 21.21 μmol/L and indirect bilirubin at 14.08 μmol/L. Parents were counseled on warning signs (feeding intolerance, recurrent fever, bleeding, poor perfusion) and scheduled follow-up.

Discharge instructions included:

i. Outpatient liver function test (total bile acids) in 1 week;
ii. Close observation of neonatal skin color, and seeking medical attention if abnormalities occur;
iii. Vitamin AD drops 1 drop daily;
iv. Recommended follow-up cardiac and cranial ultrasound at 3 months of age;
v. Recommended breastfeeding with attention to feeding safety;
vi. Attention to nutrition, warmth, regular well-child visits and vaccinations, and follow-up on psychomotor development.

A comprehensive summary of therapeutic interventions, including drug names, dosing, administration routes, oxygen modalities, and predefined weaning criteria, is provided in Table 1.

**Table 1:** Therapeutic interventions, dosing, routes, timing, and stop/weaning criteria.

Therapy/Intervention	Indication	Dose & schedule (per kg unless noted)	Route	Stop/Weaning criteria	Notes
Ampicillin + Gentamicin (empiric therapy)	Suspected early-onset sepsis due to meconium-stained fluid and respiratory distress	Ampicillin 50 mg/kg q12h + Gentamicin 4–5 mg/kg q24h	IV	Two negative cultures and decline of CRP (<10 mg/L) with stable vitals	Empiric coverage per neonatal sepsis guidelines; discontinued on day 4 to minimize antibiotic exposure
IVIG	Immunomodulation for persistent thrombocytopenia or suspected immune enhancement	1 g/kg × 1 dose	IV	Completion of planned dose and platelet recovery	Used as adjunctive support in severe cases; monitored for volume overload
Oxygen Therapy (NCPAP → nasal cannula)	Hypoxemia and respiratory distress at admission	CPAP 5 cmH₂O (FiO₂ 0.30–0.40), then weaned to nasal flow 0.5–1 L/min	NCPAP → NC	FiO₂ ≤ 0.30 and SpO₂ ≥ 94% for > 6 h without distress	Gradual de-escalation guided by respiratory rate and work of breathing; avoided prolonged positive pressure
IV Fluids (maintenance)	Limited oral intake and risk of dehydration	Dextrose/Crystalloid 60–80 ml/kg/day initially	IV	Full enteral feeds tolerated and no signs of dehydration	Maintained euvolemia to avoid capillary leak; adjusted per urine output and hematocrit
Feeds (breast milk)	Nutritional support and gut motility maintenance	On-demand breastfeeding or NG feeds if needed	PO/NG	Full oral feeding without vomiting or residuals	Early enteral feeding to preserve gut integrity and maternal-infant bonding
Acetaminophen	Fever or discomfort	10–15 mg/kg q6–8 h as needed	PO/PR	Afebrile > 24 h	Used sparingly to avoid masking clinical course
Platelet Transfusion (if required)	Platelet count <50 × 10⁹/L with bleeding tendency or invasive procedure	Per institutional threshold	IV	Hemostasis achieved	No universal cut-off for neonates; individualized decision

Additionally, a detailed daily timeline of key clinical and laboratory parameters is provided in Supplementary Table S1 to illustrate the progressive improvement in the patient's condition over the hospitalization period.

**Figure 2:**
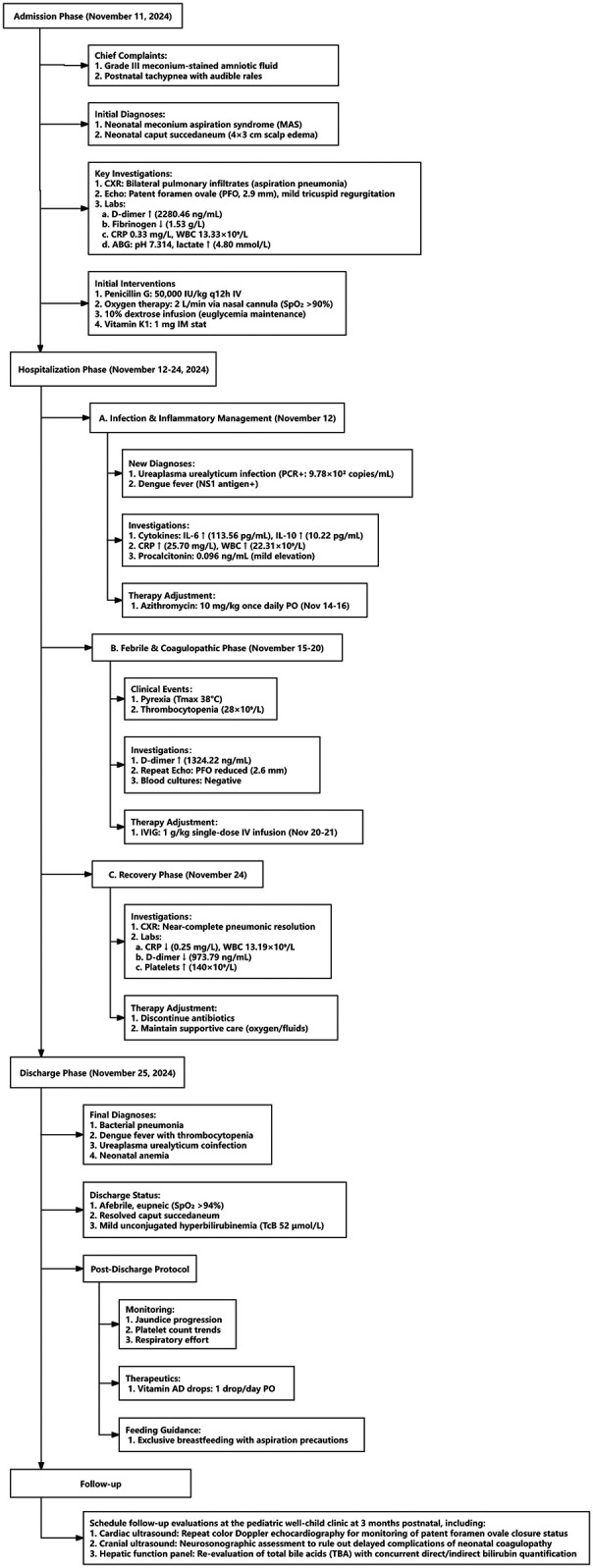
Clinical timeline of neonatal dengue with meconium aspiration.

Prognosis was determined based on sustained clinical stability (afebrile status, spontaneous breathing without oxygen support, and normal feeding), normalization of key laboratory parameters (platelet count >100 × 10⁹/L, CRP <10 mg/L, D-dimer <1,000 ng/mL), and absence of neurological or hemorrhagic manifestations during hospitalization and follow-up. A detailed overview of the patient's clinical timeline, including key diagnostic results and therapeutic interventions, is provided in Figure 2.

## Literature review

Current literature on neonatal dengue remains scarce, with most reported cases focusing on mother-to-child transmission. Due to the significant overlap in clinical presentations between neonatal dengue and other infectious diseases, early diagnosis and therapeutic intervention face substantial challenges. To situate our case within existing evidence, we acknowledge prior neonatal reports consistent with perinatal transmission, including recent case descriptions such as Mounica et al (9). To further elucidate the clinical characteristics of neonatal dengue and its mother-to-child transmission mechanisms, we conducted a comprehensive search across multiple academic databases including PubMed, Web of Science (WOS), and Embase, identifying several case reports with similarities to the current study (5, 10–16). These cases demonstrate diverse clinical manifestations, diagnostic approaches, and treatment strategies for neonatal dengue across different regions and populations (Table 2). Accordingly, our report is framed as a rare neonatal dengue case with multiple comorbidities managed at a single center, rather than a claim about age-related novelty. Through systematic analysis of these reports, we have summarized the common features of neonatal dengue in varying clinical settings and proposed potential diagnostic and therapeutic protocols. The following section presents a detailed analysis of the case data collected in this study.

## Discussion

Neonatal dengue infections represent an exceptionally rare clinical entity in global arboviral disease surveillance. World Health Organization (WHO) surveillance data indicate that neonatal cases represent only a fraction of total dengue infections, underscoring their exceptional rarity in global epidemiological reporting (17). Several factors contribute to this low incidence, including the presence of maternal antibodies, which provide neonates with passive immunity during the first few months of life. This maternal immunity, particularly when the mother is infected during pregnancy, often reduces the newborn's susceptibility to dengue virus, offering a protective barrier in the early stages of life (18). Moreover, the unique dynamics of dengue transmission, especially the relatively low occurrence of vertical transmission, further diminishes the likelihood of neonatal infections. Notably, prior reports have documented perinatal transmission resulting in neonatal disease, reinforcing the need to maintain a high index of suspicion in symptomatic neonates with maternal exposure (8).

**Table 2:** Summary of reported cases of neonatal dengue fever with maternal-fetal transmission characteristics.

Number	Source and year	Gestational age (GA)	Mode of delivery	Dengue phase	Days after birth at detection	Serum laboratory tests	Maternal symptoms and outcome	Neonatal symptoms, care, and outcome
Case 1	Pérez-Padilla et al. (2011) (10)	38w 4d	Spontaneous Vaginal	Fever phase	Day 2	Maternal and neonatal RT-PCR positive for DENV-1	Maternal fever, headache, nausea; neonatal lethargy, pallor, inability to suck, platelet count reduced to 36,000/mm^3^, received FFP and antibiotics	Neonatal platelet count reduced to 36,000/mm^3^, improved with antibiotics, safely discharged
Case 2	Sinhabahu et al. (2014) (11)	24w	Cesarean Section	Fever phase	Day 4	Maternal IgM, IgG positive, neonatal NS1 antigen strong positive, IgM weak positive	Maternal fever; neonatal sepsis, meconium aspiration syndrome; platelet reduction to 36,000/mm^3^, improved	Neonatal sepsis and meconium aspiration syndrome, treated without complications, safely discharged
Case 3	Yin et al. (2016) (12)	39w	Cesarean Section	Fever phase	Day 3	Maternal NS1 antigen positive, IgM, IgG positive, neonatal NS1 antigen positive, DENV-1 IgM, IgG negative	Maternal fever, rash, joint pain, muscle pain, frontal headache, full recovery	Neonatal fever, erythematous rash, jaundice, pneumonia, platelet reduction to 29,000/mm^3^, safely discharged
Case 4	Manzano Núñez et al. (2017) (13)	37w	Cesarean Section	Recovery phase	Day 2	Maternal NS1 antigen positive, IgM, IgG positive, neonatal NS1 antigen positive, IgG positive	Maternal fever, nosebleeds, widespread erythematous rash, treated with antibiotics	Neonatal erythematous rash, platelet reduction to 17,900/mm^3^, recovered
Case 5	Bopeththa et al. (2018) (14)	38w	Cesarean Section	Fever phase	Day 5	Maternal and neonatal NS1 antigen positive	Maternal fever, platelet drop post-surgery, required blood transfusion	Neonatal jaundice, diarrhea, fever, platelet reduction to 43,000/mm^3^, safely discharged
Case 6	Jain et al. (2019) (15)	33w 1d	Emergency Cesarean	Recovery phase	Day 6	Maternal IgM negative, later turned positive, IgG positive, neonatal NS1 antigen positive, IgM positive	Maternal muscle pain, high fever; neonatal tachypnea, mechanical ventilation, platelet reduction, eventually died due to pulmonary hemorrhage and shock	Neonatal death due to pulmonary hemorrhage and shock
Case 7	Thanh Hai Pham et al. (2023) (5)	39w 6d	Cesarean Section	Recovery phase	Day 1	Maternal and neonatal NS1 antigen positive, IgG positive	Maternal fever, rash; neonatal platelet reduction, received blood transfusion	Neonatal sepsis with dengue hemorrhagic fever, platelet reduction to 33,000/mm^3^, NICU care, safely discharged after 14 days
Case 9	Andrés Arias et al. (2024) (16)	Full-term	Cesarean Section	Fever phase	Day 6	Maternal NS1 antigen positive, IgM positive, neonatal IgM positive	Maternal fever, muscle pain, full recovery; neonatal platelet reduction, improved and discharged	Neonatal platelet reduction, recovered well, discharged on time
Case 10	Andrés Arias et al. (2024) (16)	Full-term	Cesarean Section	Recovery phase	Day 5	Maternal and neonatal NS1 antigen positive, IgG positive	Maternal fever, joint pain, platelet drop; neonatal symptoms improved, discharged	Neonatal symptoms improved, recovered well, discharged early
Case 11	Andrés Arias et al. (2024) (16)	Full-term	Spontaneous Vaginal	Recovery phase	Day 4	Maternal and neonatal IgM positive, platelet count reduced to 110,000/mm^3^	Maternal fever, fatigue, symptoms alleviated after treatment; neonatal platelet reduction, hospitalized for observation until discharge	Neonatal symptoms improved, platelet count returned to normal, safely discharged

CS, Cesarean section; d, days; ELISA, enzyme-linked immunosorbent assay; GA, gestational age; neg, negative; NICU, neonatal intensive care unit; NS1 Ag, nonstructural protein 1 antigen; PLT, platelet; pos, positive; RT-PCR, reverse transcriptase-polymerase chain reaction; VB, vaginal birth; w, weeks.

The rate of vertical transmission of dengue, although documented, remains uncommon. Studies show that while transplacental transmission is indeed possible, the incidence of neonatal dengue as a result of this route is still relatively low, with estimates suggesting that only 18.5%–22.7% of cases involving maternal viremia during the later stages of pregnancy lead to vertical transmission (5, 19). These estimates vary across studies and care settings, influenced by maternal viral load, placental integrity, and host immune factors; therefore, they should be interpreted with caution and in context (20).

Although neonatal dengue is rare, its occurrence is clinically significant due to the challenges it poses in diagnosis and management. The clinical presentation of neonatal dengue is often subtle and may overlap with other neonatal conditions such as sepsis, metabolic disturbances, and other viral infections. Symptoms, which may include fever, irritability, poor feeding, and thrombocytopenia, often mimic those of other common neonatal illnesses, making early detection difficult (16). As a result, the risk of misdiagnosis is high, and many cases go underreported or misclassified as other infections (21). The diagnostic challenges are further compounded by the absence of a definitive, easily accessible test for neonatal dengue in many settings. Laboratory markers such as NS1 antigen or serologic tests for dengue IgM and IgG may not always be sensitive in neonates, thus delaying appropriate treatment and supportive care. In this context, algorithms that integrate maternal epidemiologic exposure, neonatal timing of symptom onset, virologic testing (e.g., NS1/RT-PCR where available), and age-adjusted hematologic/coagulation parameters can improve diagnostic yield.

Furthermore, the presence of maternal dengue infection during pregnancy has been shown to correlate with adverse neonatal outcomes, including prolonged hospital stays, increased need for specialized care, and the risk of co-infections (22). This highlights the importance of early recognition and management of maternal dengue during pregnancy. Neonates born to mothers who were infected with dengue fever during the later stages of pregnancy may experience more severe disease, partly due to immune modulation induced by maternal antibodies or antibody-dependent enhancement (ADE), which can complicate the clinical course of neonatal dengue (22). ADE may lead to a more intense immune response in the neonate, exacerbating disease severity and leading to more complicated clinical presentations, including dengue hemorrhagic fever and shock syndrome (22). However, the extent to which ADE operates in individual neonatal cases remains variable, and careful interpretation alongside virologic and clinical data is warranted.

Co-infections are another critical aspect of neonatal dengue management. In many reported cases, neonatal dengue has been found to occur in conjunction with other infections, such as mycoplasma or bacterial pathogens (18, 23). These co-infections significantly worsen the prognosis and complicate treatment, as they increase the likelihood of severe outcomes like multi-organ failure, coagulopathy, and thrombocytopenia. For instance, cases of co-infection with Mycoplasma hominis have been linked to more severe presentations of neonatal dengue, where the combined effects of both infections result in plasma leakage and shock (23). In our case, the detection of Ureaplasma spp. in the upper airway was considered in the clinical context, recognizing that colonization is common and pathogenic contribution can be uncertain; management therefore balanced empiric antimicrobial therapy with ongoing reassessment.

In terms of management workflow, our case followed a structured, multidisciplinary approach aligned with neonatal sepsis and dengue management recommendations (24). The process began with early stabilization—ensuring adequate airway and oxygenation—followed by targeted laboratory testing and supportive care. Daily team huddles between neonatologists, infectious disease physicians, and nurses were used to reassess vital parameters, platelet trends, and hydration status, allowing real-time adjustment of fluid therapy. The overarching goal was to maintain euvolemia while preventing capillary leak, consistent with WHO dengue care guidelines and neonate supportive care approaches (25).

Empiric antibiotic therapy was initiated (penicillin and ampicillin–gentamicin combination) based on neonatal sepsis risk factors, including meconium-stained amniotic fluid, respiratory distress, and early thrombocytopenia. This decision was supported by existing neonatal protocols recommending short-course empiric coverage until culture results were available (26). After two sets of negative blood cultures and declining inflammatory markers (CRP <10 mg/L by day 3), antibiotics were discontinued on day 4, minimizing unnecessary antimicrobial exposure.

The diagnostic workup also reflected deliberate selection of methods based on clinical yield and sample accessibility. Initial RT-PCR was performed on maternal serum and neonatal serum to confirm vertical exposure, while respiratory PCR was used to investigate possible secondary infections. This tiered approach allowed prioritization of dengue confirmation while monitoring for bacterial or atypical pathogens—an essential consideration in resource-limited settings where simultaneous testing may not be feasible (11).

Several challenges emerged during management. First, differentiating dengue-related respiratory compromise from meconium aspiration required serial imaging and laboratory correlation, as both conditions can cause hypoxia and pulmonary infiltrates. Second, maintaining optimal fluid balance was difficult due to fluctuating hematocrit and platelet counts, risking both dehydration and fluid overload. Third, transfusion thresholds for thrombocytopenia lacked clear neonatal-specific guidance, forcing individualized clinical judgment. Finally, communication with parents—especially regarding prognosis and evolving laboratory results—was critical but time-consuming, highlighting the need for structured family communication protocols (27).

Importantly, our report does not claim age-related primacy. Instead, its contribution lies in detailing diagnostic reasoning and multidisciplinary management in a neonate with concurrent perinatal respiratory compromise and suspected vertically acquired dengue, contextualized within existing neonatal case reports (including perinatal transmission) (Mounica). This framing aligns with the need for practical guidance in endemic settings, where overlapping perinatal factors (e.g., meconium exposure) and potential copathogens frequently confound early clinical assessment.

The rarity of neonatal dengue infections is further underscored by the limited number of cases documented in the literature. Studies have shown that, while the clinical spectrum of neonatal dengue can range from asymptomatic to severe, the overall incidence remains low compared to other neonatal infections. This underscores the need for heightened awareness among healthcare providers in endemic regions to recognize the subtle signs and symptoms of dengue in newborns, especially in settings with high dengue transmission (17). A comprehensive understanding of the epidemiological context, such as the increased incidence of dengue in tropical regions during outbreak years, may help healthcare professionals better anticipate and identify at-risk newborns. Future work should also prioritize standardized reporting of neonatal cases to refine estimates of vertical transmission and clarify the role of copathogens.

From the parents' standpoint, the admission was characterized by high uncertainty about the baby's breathing, the meaning of thrombocytopenia, and whether antibiotics were necessary. They valued clear, daily updates at the bedside, including why tests (NS1/RT-PCR/serology) were ordered, what samples were used, and how results would change management. Family-centred practices—skin-to-skin contact when safe, lactation support, and stepwise de-escalation of oxygen with explicit targets (e.g., work of breathing and SpO₂ thresholds)—reduced anxiety and improved participation in care. Before discharge, counselling on warning signs (poor feeding, lethargy, bleeding, fever) and a written action plan increased caregiver confidence; parents reported that structured education and accessible contact pathways were as important as clinical recovery.

## Conclusion

In conclusion, while the overall incidence of neonatal dengue remains low, the increasing frequency of dengue outbreaks worldwide, especially in endemic regions like Southeast Asia and South America, makes it essential for healthcare systems to remain vigilant. Neonatal dengue poses significant diagnostic challenges, often presenting with nonspecific symptoms that overlap with other neonatal conditions, thus making early detection and management difficult. Future research should focus on improving diagnostic tools, understanding the immunological mechanisms underlying neonatal dengue, and developing preventive strategies for high-risk populations, such as pregnant women in endemic areas. Additionally, a better understanding of the role of maternal immunity and co-infections in the pathogenesis of neonatal dengue will be critical in improving clinical outcomes for affected newborns. Equally, routine incorporation of family-centred communication (daily updates, discharge education, and clear escalation plans) should be emphasized, as it directly addresses caregiver concerns and may improve adherence and post-discharge safety.

## Data Availability

The datasets presented in this study can be found in online repositories. The names of the repository/repositories and accession number(s) can be found in the article/Supplementary Material.
