# Supplementary material for: Perinatal dengue in a neonate with multiple comorbidities: case report and literature review
Source: Front Pediatr. 2025 Oct 27;13:1685280. doi: 10.3389/fped.2025.1685280 (PMC12598494; doi:10.3389/fped.2025.1685280)
Supplement: Supplementary file 1 [file Table1.docx]

Supplementary Table S1. Daily Timeline of Key Clinical and Laboratory Parameters Showing Progressive Improvement

| **Date (Hospital Day)** | **Body Temperature (°C)** | **Respiratory Rate (breaths/min)** | **Platelet Count (×10⁹/L)** | **CRP (mg/L)** | **D-dimer (ng/mL)** | **Key Events/Interventions** | **Clinical Status** |
| --- | --- | --- | --- | --- | --- | --- | --- |
| Nov 11 (Day 1) | 36.6 | 56 | 137 | 0.33 | 2280 | Admission; penicillin, oxygen started; NS1 testing ordered | Tachypnea, rales; metabolic acidosis |
| Nov 12 (Day 2) | 36.8 | 50 | 117 | 25.7 | - | NS1 positive; Ureaplasma detected; azithromycin added | Inflammatory response increased; stable hemodynamics |
| Nov 15-19 (Days 5-9) | 37.5-38.0 (peak 38) | 45-50 | 50-80 (declining) | 10-20 | 1324 | Fever onset; monitoring intensified | Fever, thrombocytopenia worsening; no bleeding |
| Nov 20 (Day 10) | 37.8 | 45 | 29 (nadir) | 15.0 | 1324 | IVIG 1g/kg given; oxygen reduced to 1L/min | Thrombocytopenia peak; immune modulation |
| Nov 24 (Day 14) | 36.5 | 40 | 140 | 0.25 | 974 | Antibiotics discontinued; chest X-ray improved | Significant recovery; afebrile, reduced respiratory effort |
| Nov 25 (Discharge) | 36.6 | ＜60 (spontaneous) | 150+ | <1.0 | <1000 | Discharge with follow-up | Stable; no fever/respiratory distress; normal feeding |

Notes: Data derived from serial monitoring. Improvement evident in declining CRP, recovering platelets, and stabilizing vitals post-Day 10. "-" indicates not tested that day.
